# Prevalence of musculoskeletal pain among undergraduate students

**DOI:** 10.3389/fmed.2024.1403267

**Published:** 2024-09-20

**Authors:** Geetha Kandasamy, Mona Almanasef, Tahani Almeleebia, Khalid Orayj, Eman Shorog, Asma M. Alshahrani, Kousalya Prabahar, Vinoth Prabhu Veeramani, Palanisamy Amirthalingam, Saleh F. Alqifari, Fayez Alrashidi, Muteb Aldurum, Faiz Almutiri, Adel Alzaidi, Fahad Almutairi

**Affiliations:** ^1^Department of Clinical Pharmacy, College of Pharmacy, King Khalid University, Abha, Saudi Arabia; ^2^Department of Clinical Pharmacy, College of Pharmacy, Shaqra University, Dawadimi, Saudi Arabia; ^3^Department of Pharmacy Practice, Faculty of Pharmacy, University of Tabuk, Tabuk, Saudi Arabia; ^4^Ministry of Health, King Abdullah Hospital, Bisha, Saudi Arabia; ^5^Ministry of Health (MOH), Dhurma Hospital, Dhurma, Saudi Arabia; ^6^Ministry of Health (MOH), Huraymila Hospital, Huraymila, Saudi Arabia

**Keywords:** low-back pain, shoulder pain, musculoskeletal disorders, neck pain, students

## Abstract

**Introduction:**

Musculoskeletal disorders (MSDs) are rapidly rising in Saudi Arabia, reaching levels similar to those in the Western world. Hence, we aimed to assess the prevalence of neck, shoulder, and lower back pains (musculoskeletal pain, MSP) among students at King Khalid University in Abha, Saudi Arabia.

**Methods:**

This cross-sectional study was conducted at King Khalid University in Abha, Saudi Arabia, from March 2023 to August 2023. Inclusion criteria were: university students aged 18 years and older of both sexes who agreed to participate in the study. The modified Nordic questionnaire was used, which comprised three parts.

**Results:**

Out of 536 respondents, 337 were women and 199 were men. The average body mass index (BMI) of the study population was 25.3 ± 4.01. In total, 223 (41.60%) had a history of MSDs. Only 232 (43.28%) of the population did regular exercise. According to multiple logistic regression analysis, factors associated with MSDs are mobile device use (with both hands) with a large neck tilt below the horizon line position (OR = 2.276, CI 1.178–4.397, *p* = 0.014), family history of trauma (OR = 5.450, 95% CI 3.371–8.811, *p* = 0.000), family history of MSDs (OR = 4.241, 95% CI 2.296–7.835, *p* = 0.000), coffee consumption (OR = 1.967, CI 1.281–3.020, *p* = 0.002), and time spent on electronic devices: 1–3 h (OR = 0.252, 95% CI 0.124–0.511, *p* = 0.0001), 4–6 h (OR = 0.455, 95% CI 0.237–0.873, *p* = 0.018), and 6–9 h (OR = 0.348, 95% CI 0.184–0.660, *p* = 0.001).

**Conclusion:**

The present study concludes that MSP among university students is high. A history of trauma, a family history of MSDs, the hand and neck position when using electronic devices, the amount of time spent using them, and regular exercise are risk factors that are strongly associated with MSP. There is strong evidence to suggest that increasing physical activity plays a significant role in enhancing the functionality of the musculoskeletal (MSK) system and alleviating pain. It is recommended that universities implement educational programs to raise awareness and health screenings about the impact of device usage on MSK health and the benefits of regular exercise.

## 1 Introduction

A growing number of people are experiencing neck pain (NP), which has a significant socioeconomic impact on people, their families, and communities. NP is a significant contributor to illness, lower educational achievement, and missing university classes, all of which have an impact on students’ future jobs. Musculoskeletal disorders (MSDs) are characterized as injuries to the musculoskeletal (MSK) system that can result from repeated or isolated trauma and that negatively impact a person’s day-to-day activities (1). In Saudi Arabia, rates of musculoskeletal pain (MSP) are developing quickly and are now comparable to those in the West: MSP accounts for 38% of visits to family practice and is the third most common reason for hospital visits (2). Neck discomfort is regarded as the fourth most common cause of disability because it has an annual prevalence rate of >30%. Although acute NP often resolves with or without therapy, approximately 50% of individuals will experience pain recurrence to some extent (3).

In the past 30 years, there has been an increase in the prevalence of neck–shoulder pain (NSP) and low back pain (LBP) among teenagers. The use of computers and mobile phones for extended periods of time is one aspect that has contributed to this rise. The lower back is the third most typical area of non-traumatic pain in adolescents of both sexes, with the neck and shoulder being the most frequently affected musculoskeletal regions (4). According to several studies, the most frequent risk factors for LBP include female sex, advanced age, hyperactivity, competitive sports, decreased life quality, and emotional symptoms (5). LBP is as common as neck discomfort, with a prevalence of 7.6%. Although many individuals have acute self-limited LBP that does not require medical attention, people who experience LBP earlier in adolescence are more likely to experience it later in life (6). Psychological elements, such as depression and psychosomatic symptoms, have also been proposed as risk factors for MSP and are related to a lower quality of life (7). The spread of NSP is further accelerated by psychological issues and poor self-reported health (8). The development of mobile technology in the 21st century has connected an increasing number of people daily via their phones. They spend more time on social media, cell phones, tablets, text reading, and other electronic devices, which causes their necks to flex for an extended period of time and results in text neck syndrome (9). This is due to repeated, intense tension on the flexed neck. It causes headaches (10) as well as pain in the neck, shoulders, and head. It is a growing health concern, and the youth may be more affected than older generations.

Uncomfortable postures, non-stop smartphone and computer use, and frequent or prolonged laptop use have all been identified as risk factors for musculoskeletal problems (11). For instance, a systemic evaluation of studies of the working population in Europe from 2010 found that 25% of people suffer from NSP (12). A Canadian study on users of mobile devices found that 68% of participants had neck complaints and 46–52% of individuals experienced shoulder symptoms (13). A total of 40% of participants in a Chinese study on young phone users reported having NSP (14).

The relationship between texting on mobile devices and NSP has been the subject of numerous studies. Prolonged neck flexion is also associated with pain in the neck, shoulder, and upper extremities during other activities (15). This can be explained by the static muscle load brought on by extended neck flexion, the lack of arm support, and the repetitive motion of the fingers, especially when just one hand is being used (16). The user’s position when using a mobile phone is another factor taken into account. Everyone agrees that sitting with a straight neck and supporting forearms is ideal. This position should only be held for a brief period of time, along with holding the cell phone with both hands and utilizing both thumbs. The primary determinants of NSP and its intensity are the frequency of cell phone usage, the reason for using a cell phone, the degree of neck flexion while using a cell phone, and body position (17).

However, further research is required to determine how these and other factors affect university students. Hence, the purpose of our study is to assess the prevalence of neck, shoulder, and lower back MSP among students at King Khalid University in Abha, Saudi Arabia.

## 2 Methods

### 2.1 Study design and sample size

This cross-sectional study was conducted at King Khalid University in Abha, Saudi Arabia, between 2 March and 30 August 2023. The sample size was calculated using the Raosoft (18) sample size calculator (Cochran’s formula). The sample size was based on the total number of students in the university (1,500) with a 95% confidence level, a 50% response rate, and a 5% margin of error, according to Raosoft’s online sample size calculator. The target sample size was set at *n* = 385 to reduce the error in the results and improve the reliability of the study. A total of 588 students responded with a completed questionnaire. Of these 588 questionnaires, 52 were excluded due to insufficient data. The effect size was found to be 0.3 when the df (degrees of freedom) was as high as 5 (between two variables of interest) and the power of the study was 0.80.

### 2.2 Inclusion criteria

University students of both sexes who were at least 18 years old and willing to participate in the study met the inclusion criteria. Exclusion criteria were participants younger than 18 years and non-university students. Incomplete questionnaires were also excluded.

### 2.3 Questionnaire

The Standardized Nordic Questionnaire (19), which was translated into Arabic for use in this study, was modified into an online self-administered questionnaire that was delivered via Google Forms. The modified Nordic questionnaire was used according to the method outlined by Smith et al. (20). The internal consistency of the questionnaire items was measured using Cronbach’s alpha to evaluate reliability. The Cronbach’s alpha coefficient was 0.70.

The questionnaire comprised three parts. Part A consisted of questions on sociodemographic information: sex, age, weight, height, BMI, and study year. BMI was calculated by the following formula: weight (kg)/height (m)^2^. Overweight and obesity were defined by BMI values of 25 and 30 kg/m^2^, respectively (21).

Part B consisted of questions related to risk factors, namely smoking habits, exercise habits, coffee consumption, history of trauma, and family history of musculoskeletal diseases; type of device, purpose, and length of use; hours spent using a computer or studying each day; position of the neck and hands when using a smartphone; severity of neck or shoulder pain experienced while using an electronic device; use of painkillers; performing neck and shoulder exercises after using an electronic device for an extended period of time; and relationship between pain and prior injury and how it affects daily activities.

In Part C, questions were asked about neck, shoulder, and lower back pain in the previous 7 days and 12 months.

### 2.4 Data collection

The web link to the questionnaire was distributed to the students of all academic years using Google Forms, through social media such as WhatsApp groups, Twitter, Facebook, and email. Written informed consent was obtained by explaining the purpose of the study to the participants and assuring them of anonymity and confidentiality.

Participants were told that they had the right to decline the survey questionnaire at any time. Incomplete responses were excluded from the data analysis. The Institutional Review Board of King Khalid University ECM 2023-706 gave the study its approval, and the study was carried out in accordance with the Declaration of Helsinki.

### 2.5 Statistical analysis

The Statistical Package of Social Sciences (SPSS) program, version 17, was used for the analysis. The frequencies, percentages, means, and SDs were obtained using descriptive analysis. A chi-squared test at *p* < 0.05 was used to evaluate the association between the dependent variables and the independent variables. The odds ratio was calculated with a 95% confidence interval in order to assess the strength of the association. Multivariable logistic regression analysis was used to examine the relationship between the independent variables and the dependent variables (MSDs in the previous 7 days in at least one location and MSDs in the previous 12 months in at least one location). The effect size was found to be 0.3 when the df (degrees of freedom) was as high as 5 (between two variables of interest) and the power of the study was 0.80.

## 3 Results

A total of 588 people took part in this survey, with 52 surveys not complete and therefore excluded. Out of *n* = 536 participants, 337 (62.87%) were women, and 199 (37.13%) were men. The average age of the participants was 23.72 ± 2.86 years. The average BMI of the study population was 25.3 ± 4.01 kg/m^2^. The male participants were more likely to smoke than their female counterparts, with male smokers (*n* = 150) accounting for 27.98% of the total population and female smokers (*n* = 37) accounting for 6.90%. The academic years of the participants were as follows: 33 first years (6.2%), 47 s years (8.8%), 92 third years (17.2%), 186 fourth years (34.7%), and 178 fifth years (33.2%). The time spent on devices was distributed as follows: 114 (21.27%) used devices between 1 and 3 h per day, 158 (29.28%) between 4 and 6 h, 172 (32.09%) between 6 and 9 h, and 92 (17.16%) over 9 h per day. A total of 295 people (55.04%) reported using their electronic devices for studying, whereas 241 (44.96%) reported using them for entertainment and social media. A total of 223 (41.60%) had a history of neck, shoulder, or lower back injuries, while 313 (58.40%) had no such history. A total of 112 people (20.90%) had a family history of MSDs, compared to 424 (79.10%) who did not. Only 232 (43.28%) of the population did regular exercise, whereas 304 (56.72%) did not. At least three cups of coffee per day were consumed by 332 (61.94%) of the population, more than half, compared to 204 (38.06%), who consumed less than three cups per day. In total, 147 (27.43%) of the population used iPads or tablets and smartphones, followed by 103 (19.22%) who used smartphones, 84 (15.67%) who used iPads or tablets, smartphones, and computers, 71 (13.25%) who used iPads or tablets, 62 (11.57%) who used iPads or tablets and computers, 41 (7.65%) who used smartphones and computers, and 28 (5.22%) who used computers. A total of 75 (14%) used their device with both hands with a slight tilt of the neck below the horizon line, 159 (29.7%) used their device with both hands with a large neck tilt below the horizon line, 144 (26.9%) used their device with one hand with a large neck tilt below the horizon line, and 158 (29.5%) used their device with one hand with a slight tilt of the neck below the horizon line (Table 1).

**Table 1:** Sociodemographic characteristics of the study population.

Variables	Category	*N* (536) %
Sex	Male	199 (37.13)
	Female	337 (62.87)
Academic Year	1	33 (6.2)
	2	47 (8.8)
	3	92 (17.2)
	4	186 (34.7)
	5	178 (33.2)
BMI (kg/m^2^)	25.3 ± 4.01	
History of trauma (neck/shoulder/low back):	Yes	223 (41.60)
	No	313 (58.40)
Family history of MSDs:	Yes	112 (20.90)
	No	424 (79.10)
Regular exercise	Yes	232 (43.28)
	No	304 (56.72)
Coffee	Coffee ≥3 cups consumed per day	332 (61.94)
	Coffee ≤3 cups consumed per day	204 (38.06)
Position of neck and hands when using the device	Mobile use (with both hands) with a slight tilt of the neck below the horizon line	75 (14)
	Mobile use (with both hands) with a large neck tilt below the horizon line	159 (29.7)
	Mobile use (with one hand) with a large neck tilt below the horizon line	144 (26.9)
	Mobile use (with one hand) with a slight tilt of the neck below the horizon line	158 (29.5)
Time spent using the device	1–3 h	114 (21.27)
	4–6 h	158 (29.28)
	6–9 h	172 (32.09)
	9 h and more	92 (17.16)

Prevalence of MSD body sites: 354 students (66.04%) reported having NP in the previous 7 days compared to 308 (57.46%) who did not. In the previous 12 months, 228 students (42.54%) reported NP, compared to 229 (42.72%) who did not. A total of 327 people (61.01%) reported having lower back discomfort in the past week, compared to 297 (55.41%) who did not. A total of 239 people (44.59%) reported having lower back pain in the previous 12 months, compared to 297 (55.41%) who did not. A total of 307 people (57.28%) reported having shoulder pain in the previous 7 days, compared to 288 (53.73%) who did not. A total of 248 people (46.27%) reported having shoulder pain in the previous 12 months, compared to 209 (38.99%) who did not.

Using chi-squared tests, a significant association was found for those with MSDs during the previous 7 days among those who had a history of trauma (neck/shoulder/lower back pain) compared to those who had no trauma (OR = 3.46, 95% CI 2.08–5.73, *p* = 0.0001), among those who reported a significant family history of MSDs in comparison to those who did not (OR = 2.72, 95% CI 1.40–5.28, *p* = 0.0030), among those who did regular exercise in comparison to those who did not (OR = 0.56, CI 0.36–0.86, *p* = 0.0082), and among those with certain positions of their neck and hands when using their device (*p* = 0.0003).

For those who had MSDs during the previous 12 months, there was a significant association among those who had a history of trauma (neck/shoulder/lower back pain) compared to those who had no trauma (OR = 3.89, 95% CI 2.59–5.86, *p* = 0.0001) and among those who reported a significant family history of MSDs in comparison to those who did not (OR = 3.93, 95% CI 2.24–6.92, *p* = 0.0001). The prevalence of MSDs in the past year showed a strong association in the regular exercise group compared to those who were not in the group (OR = 1.48, CI 1.03–2.14, *p* = 0.03). The prevalence of MSDs in the past year was higher among the students in the coffee consumption group compared to those who were not in the group (OR = 3.29, CI 2.28–4.74, *p* = 0.0001). In the past year, the prevalence of MSDs showed an association among the students with the time spent on the used device (*p* = 0.04) (Table 2).

**Table 2:** Factors associated with MSDs in the previous week and previous 12 months among students using the chi-squared test.

Variables	Category	MSDs during the previous 7 days				MSDs during the previous 12 months			
		Yes	No	OR (95% CI)	*p*-Value	Yes	No	OR (95% CI)	*p*-Value
Sex	Male	167	32	1.51 (0.96–2.39)	0.07	131	68	1.01 (0.69–1.46)	0.95
	Female	261	76			221	116		
Academic year	First Year	27	6		0.06	19	14		0.79
	Second Year	40	7			32	15		
	Third Year	69	23			63	29		
	Fourth Year	159	27			124	62		
	Fifth Year	133	45			114	64		
Body mass index (kg/m^2^)		428 (Mean, IQR) 24.90, 2.30	108 (Mean, IQR) 24.60, 2.23			352 (Mean, IQR) 25.30, 2.00	184 (Mean, IQR) 24.60, 2.00		
History of trauma (neck/shoulder/low back):	Yes	201	22	3.46 (2.08–5.73)	*0.0001	183	40	3.89 (2.59–5.86)	*0.0001
	No	227	86			169	144		
Family history of MSDs	Yes	101	11	2.72 (1.40–5.28)	*0.0030	96	16	3.93 (2.24–6.92)	*0.0001
	No	327	97			256	168		
Regular exercise	Yes	173	59	0.56 (0.36–0.86)	*0.0082	164	68	1.48 (1.03–2.14)	*0.03
	No	255	49			188	116		
Coffee	Coffee ≥3 cups consumed per day	266	66	1.04 (0.67–1.61)	0.84	233	99	3.29 (2.28–4.74)	*0.0001
	Coffee ≤3 cups consumed per day	162	42			85	119		
Position of neck and hands when using the device	Mobile use (with both hands) with a slight tilt of the neck below the horizon line	49	26		*0.0003	50	25		0.88
	Mobile use (with both hands) with a large neck tilt below the horizon line	140	19			102	57		
	Mobile use (with one hand) with a large neck tilt below the horizon line	119	25			98	46		
	Mobile use (with one hand) with a slight tilt of the neck below the horizon line	120	38			102	56		
Time spent using the device	1–3 h	90	24		0.09	68	46		*0.04
	4–6 h	124	34			106	52		
	6–9 h	147	25			107	65		
	9 h and more	67	25			71	21		

*p < 0.05 considered significant.

With multiple logistic regression analysis, factors associated with MSP during the past week were being fourth-year students (OR = 2.122, 95% CI 1.160–3.879, *p* = 0.015), having a family history of MSDs (OR = 4.273, 95% CI 2.413–7.567, *p* = 0.000), doing regular exercise (OR = 0.56, CI 0.36–0.86, *p* = 0.001), being in the coffee consumption group (OR = 1.687, CI 1.020–2.791, *p* = 0.042), and using a mobile device with both hands with a large neck tilt below the horizon line position (OR = 2.276, CI 1.178–4.397, *p* = 0.014). Factors associated with MSP during the past year were being male (OR = 0.520, 95% CI 0.325–0.832, *p* = 0.006), being a fourth-year student (OR = 1.711, 95% CI 1.025–2.856, *p* = 0.040), having a family history of trauma (OR = 5.450, 95% CI 3.371–8.811, *p* = 0.000), having a family history of MSDs (OR = 4.241, 95% CI 2.296–7.835, *p* = 0.000), being in the coffee consumption group (OR = 1.967, CI 1.281–3.020, *p* = 0.002), and spending the following time on devices: 1–3 h (OR = 0.252, 95% CI 0.124–0.511, *p* = 0.000), 4–6 h (OR = 0.455, 95% CI 0.237–0.873, *p* = 0.018), and 6–9 h (OR = 0.348, 95% CI 0.184–0.660, *p* = 0.001) (Table 3).

**Table 3:** Multiple logistic regression analysis of factors associated with MSDs in the previous 7 days and previous 12 months.

Variables	Category	MSDs during the previous week						MSDs during the previous 12 months					
		Yes	No	B value	*p*-Value	OR	(95% CI)	Yes	No	B value	*p*-Value	OR	(95% CI)
Sex	Male	167	32	0.191	0.491	1.210	(0.703–2.083)	131	68	−0.654	*0.006	0.520	0.325–0.832
	Female	261	76					221	116				
Academic year	First Year	27	6	0.372	0.497	1.450	0.496–4.240	19	14	−0.304	0.498	0.738	0.306–1.779
	Second Year	40	7	−0.166	0.736	0.847	0.322–2.225	32	15	−0.211	0.610	0.810	0.360–1.821
	Third Year	69	23	−0.163	0.631	0.850	0.438–1.649	63	29	0.523	0.110	1.687	0.889–3.204
	Fourth Year	159	27	0.752	*0.015	2.122	1.160–3.879	124	62	0.537	*0.040	1.711	1.025–2.856
	Fifth Year	133	45					114	64				
Body mass index (kg/m^2^)				0.016	0.598	1.016	0.957–1.079			0.051	0.063	1.052	0.997–1.110
History of trauma (neck/shoulder/low back):	Yes	201	22	1.452	*0.000	4.273	2.413–7.567	183	40		*0.000	5.450	3.371–8.811
	No	227	86					169	144				
Family history of MSDs	Yes	101	11	0.688	0.057	1.990	0.980–4.041	96	16	1.445	*0.000	4.241	2.296–7.835
	No	327	97					256	168				
Exercise	Yes	173	59	−0.844	*0.001	0.430	0.265–0.698	164	68	0.238	0.268	1.269	0.833–1.934
	No	255	49					188	116				
Coffee	Coffee ≥3 cups consumed per day	266	66	0.523	*0.042	1.687	1.020–2.791	233	99	0.677	*0.002	1.967	1.281–3.020
	Coffee ≤3 cups consumed per day	162	42					85	119				
Position of neck and hands when using the device	Mobile use (with both hands) with a slight tilt of the neck below the horizon line	49	26	−0.401	0.243	0.669	0.341–1.313	50	25	0.376	0.271	1.456	0.746–2.842
	Mobile use (with both hands) with a large neck tilt below the horizon line	140	19	0.823	*0.014	2.276	1.178–4.397	102	57	0.152	0.577	1.165	0.681–1.991
	Mobile use (with one hand) with a large neck tilt below the horizon line	119	25	0.314	0.316	1.369	0.741–2.529	98	46	−0.059	0.833	0.943	0.546–1.629
	Mobile use (with one hand) with a slight tilt of the neck below the horizon line	120	38					102	56				
Time spent on using the device	1–3 h	90	24	−0.060	0.872	0.942	0.454–1.953	68	46	−1.380	*0.000	0.252	0.124–0.511
	4–6 h	124	34	−0.127	0.709	0.881	0.452–1.715	106	52	−0.787	*0.018	0.455	0.237–0.873
	6–9 h	147	25	0.577	0.095	1.780	0.904–3.507	107	65	−1.056	*0.001	0.348	0.184–0.660
	9 h and more	67	25					71	21				

B, Beta value; OR, odds ratio; CI, confidence interval, *p < 0.05 considered significant.

## 4 Discussion

A BMI of 25.3 kg/m^2^ indicates overweight in the study population. According to the authors of one prior study, the general adult population is more likely to experience chronic pain in the lower back, neck, and shoulders as a result of physical inactivity and high BMI (22). Another study found that overweight and obese young individuals are in the risk category for mechanical NP and different cervical diseases, and it is crucial to raise awareness of preventive measures such as posture correction exercises and weight management techniques (23). Yogasana relieves tense and exhausted limbs by restoring retracted and stiff muscles. The practice of specific asanas is a potent tool for the prevention or treatment of MSDs, including postural problems, forward head posture, chronic neck tension, depressed chest, carpal tunnel syndrome, impingement syndromes, outlet syndrome, subacromial pain syndrome, and spinal disk pathologies (24).

In this study, the time that participants spent on their devices was distributed as follows: 114 (21.27%) spent between 1 and 3 h per day, 158 (29.28%) spent between 4 and 6 h, 172 (32.09%) spent between 6 and 9 h, and 92 (17.16%) spent more than 9 h. More than half the study population, 55.04%, said they used their electronic devices for learning, while 44.96% said they used them for social media and entertainment.

Another study found that approximately 39% of participants watched cartoons or movies on their devices, 27% used social media, and 17% played video games. Due to the COVID-19 epidemic at the time of the study, 24.48% of participants also used these devices to take online courses. Only a small percentage of participants (8.74%) used mobile devices for routine communication (25). An Asian study that supports this idea showed that social influences from various online activities, such as social media, online classes, and gaming, have a significant impact on developing a preference for technology use or internet addiction and displaying disinterest in outdoor activities (26).

According to another previous study, 87.5% of participants use digital devices regularly. They all have cell phones and 89.2% of them have tablets. A total of 70.0% of participants said they used digital devices while lying in bed, and 60% said they used computers for less than 6 h per day, phones for less than 10 h per day, and other digital devices for less than 3 h per day (27).

We discovered that the majority of students frequently use apps on mobile devices. Utilizing digital devices for longer periods of time increases the chance of health problems. The various levels of education rely on online learning using computers, smartphones, tablets, iPads, and other devices, especially in the wake of the COVID-19 pandemic. Students have many concerns about using these digital devices since they may cause physical discomfort, particularly neck and back pain. According to a study by Cheung et al. (28), 46.3% of students used computers less than an hour a day in 2022.

The majority of the individuals in our study reported regular use of mobile digital devices, which raises their risk of neck and back pain. A total of 41.60% had a history of neck, shoulder, or lower back injuries, while 58.40% of the population had no such history. Only 20.90% had a family history of MSDs. Alshagga et al. (29) concluded that those who had a family history of MSDs with trauma to the shoulder, neck, or lower back, were at a high risk of developing MSP, supporting the finding in our study that there is a substantial correlation between MSP and a history of trauma (30). Our study reported that only 43.28% of the population had a regular exercise habit. A previous study concluded that pericervical strength and range of motion in young adults were improved by a 6-week pericervical muscle stretching and strengthening program. The majority of subjects experienced reduced cervical pain (31). According to the meta-analysis, there is a statistically significant difference favoring strengthening training over no exercise for pain relief (32).

In our study, 61.94% (*n* = 332), or more than half the population, consumed more than three cups of coffee per day. Caffeine, a component of coffee, helps fight stress, tiredness, and pain. McPartland and Mitchell (33) noted significant caffeine consumption among patients with lower back pain and emphasized the need to limit coffee drinking in this population since caffeine raises urine calcium levels and may have long-term negative effects on bones. Interestingly, prolonged excessive coffee consumption produced a vicious cycle between shoulder and NP and short sleep cycles. The findings suggest that people who get less than 6 h of sleep each night or who experience shoulder and NP should limit their intake of coffee to two cups per day (34).

In the present study, 27.43% of students reported using iPads or tablets and smartphones, and 19.22% reported using smartphones. A previous study found that 45% of students used smartphones, followed by smartphones and computers (19.2%) (35). In a previous study, 71.2% of respondents reported cervical pain as their most frequent symptom. The use of a cell phone can lead to improper body mechanics and posture, which can result in pain in the neck, shoulders, upper back, arms, and throughout the entire body. The uncomfortable and repetitive stress injury caused by extensive and prolonged use of cell phones is referred to in medicine as text neck syndrome. The majority of cell phone users are known to be impacted by this condition, which is also perceived as an increasingly widespread worldwide burden affecting individuals of all ages and genders who are a part of every community. This syndrome is the result of repeated stress to the body from using handheld electronic devices over an extended period of time, specifically repeated forward head flexion while looking at cell phone screens for an extended period (36). A study was done to look into the stresses experienced by the spine when the head is bowed forward into a worsening position. The results of the investigation demonstrated that there is a significant increase in weight on the spine with different degrees of head flexion. Significantly additional stress is placed on the cervical spine as a result of the loss of the spine’s natural curve (37).

In the current study, 195 (36.38%) of participants used a mobile device while holding it in one hand and tilting their necks below the horizon line. Our findings are consistent with other research: 40.5% of users had their necks slightly tilted downward while using a mobile device (35). MSP in the neck and shoulders has been linked to the position of the head and neck when using electronic devices (9). In the current study, more participants used their cell phones with one hand while bending their necks well below the horizon line than did so in any other way. The prevalence of MSDs among medical students was shown to be significant in another study; 85.3% of the students had MSDs in at least one site at any given moment (30). There is a considerable risk of developing new health issues when using equipment for a long period in the same position. The amount of time spent utilizing electronics and musculoskeletal discomfort are significantly correlated. Raising the amount of time spent using electronics would result in more musculoskeletal injuries. Past research found that young, healthy college students who use their smartphones excessively experience NP (38). According to previous research, neck discomfort complaints increased in direct proportion to the amount of time spent using electronic devices, with a close correlation between the two (39).

In the current study, in terms of MSD site, 354 students (66.04%) reported having NP in the previous week, 327 (61.01%) reported having lower back pain, and 307 (57.28%) reported having shoulder pain. In another study, the prevalence over the previous 7 days was 60.64%, with the upper and lower back (31.91%) neck (21.28%), and shoulders (15.96%) being the most prevalent regions (40). According to Smith et al. (41), 67.6% of Chinese medical students experienced pain in the previous 7 days, with lower back pain accounting for the majority of cases (20.8%), followed by knee and NP (12.1%). The neck (55.8%) was reported as the body part experiencing the most pain from smartphone use in a second Korean survey of smartphone users by Kim et al. (42). Similarly, Namwongsa et al. (43) found that the most common musculoskeletal condition among smartphone users in Thailand was NP. Furthermore, the cross-sectional investigations found that mobile touchscreen device users had the highest prevalence rates of neck and/or shoulder problems, ranging from 26.3 to 60%. According to earlier research, uncomfortable postures are a physical risk factor for neck MSK disease in employees. Many musculoskeletal issues can result from prolonged smartphone use (44). Particularly when using a smartphone, uncomfortable postures may be encouraged.

In the previous 12 months, 42.54% (*n* = 228) of students reported NP, 44.59% (*n* = 239) reported having LBP, and 46.27% (*n* = 248) reported having shoulder pain. Another study found that the prevalence of NSP and LBP among adolescents is 69.4 and 62.2%, respectively (4). Self-reported LBP and NSP were already very common among teenagers, according to a previous study. Girls are more likely to report LBP and NSP. Yue et al. discovered that there is a significant association between the risk of LBP and each of the following: daily computer use (OR = 1.32, 1.05–1.60), daily mobile use (OR = 1.32, 1.00–1.64), and daily TV watching (OR = 1.07, 1.04–1.09). We reported a linear correlation between LBP and daily computer use, with an 8.2% increase in LBP for each hour of use (45). A previous study revealed that a large percentage of medical students have MSP. Depressive and psychosomatic symptoms, in addition to a history of trauma, are factors that raise the risk of MSP (46). This study has limitations. Because it is a single-institution study, non-probability sampling reduces the representativeness of the sample by not guaranteeing that each person has an equal chance of being chosen. Thus, generalizing the study’s findings is not possible.

## 5 Conclusion

The present study concludes that MSP among university students is high. A history of trauma, family history of MSDs, hand and neck position when using a device, amount of time spent using it, and regular exercise are risk factors that are strongly associated with MSP. There was a strong correlation between NSP and LBP and certain individual, ergonomic, and occupational variables. To provide a successful preventive strategy for these extremely common and undetected conditions, research on other interventional strategies is also necessary. There is strong evidence to suggest that increasing physical activity plays a significant role in enhancing the functionality of the musculoskeletal system and alleviating pain. It is recommended that universities implement educational programs to raise awareness and health screenings on the impact of device usage on MSK health and the benefits of regular exercise.

## Data Availability

The original contributions presented in the study are included in the article/supplementary material, further inquiries can be directed to the corresponding author.
